# Traumatic Childbirth and Post Traumatic Stress Disorder: prevalence in a Brazilian cohort

**DOI:** 10.1192/j.eurpsy.2024.768

**Published:** 2024-08-27

**Authors:** F. D. L. Osório, M. L. Baldisserotto, M. M. Theme Filha, S. Ayers

**Affiliations:** ^1^São Paulo University, Ribeirão Preto; ^2^Escola Nacional de Saúde Pública Sérgio Arouca (ENSP/ Fiocruz), Rio de Janeiro, Brazil; ^3^City - University of London, London, United Kingdom

## Abstract

**Introduction:**

Although birth is experienced, in most cultures, as a positive event, for a significant percentage of women, it is considered a traumatic event, which can be associated with the development of psychopathologies, with negative impacts for the mother and the baby .

**Objectives:**

As part of a larger, multicenter study called Intersect, we aim to assess the prevalence of women who considered childbirth traumatic, in a cohort of women in southeastern Brazil, and the association with the outcome of post-traumatic stress disorder (PTSD).

**Methods:**

A total of 427 women who gave birth in two hospitals in southeastern Brazil in the period from May to October 2022 were included in the study, who answered self-assessment instruments, through on a telephone interview, in the period from 6 to 12 postpartum weeks. For the purposes of this study, the City Birth Trauma Scale stands out.

**Results:**

The participants had a mean age of 28.4 (± 6.4) years, 39.2% were primiparous and 76.1% had a partner. The results showed that 51.3% of them considered the birth moderately or extremely traumatic (N=218). Of these, 50.9% met criterion A for PTSD according to the DSM-5 (N=111) and among these, 20.7% had a PTSD profile (N=23; City-Birth >28 points). These mothers represent 5.4% of the total sample.

**Conclusions:**

there is a high prevalence of traumatic experiences during childbirth, with high rates of PTSD associated with this condition, which requires attention from the medical community in order to track and treat PTSD associated with birth and, from the public authorities, in the institution of preventive measures, through public policies aimed at this population.

**Disclosure of Interest:**

None Declared

